# Analysis of Heat Transfer Characteristics of a GnP Aqueous Nanofluid through a Double-Tube Heat Exchanger

**DOI:** 10.3390/nano11040844

**Published:** 2021-03-25

**Authors:** Uxía Calviño, Javier P. Vallejo, Matthias H. Buschmann, José Fernández-Seara, Luis Lugo

**Affiliations:** 1 Grupo GAME, Departamento de Física Aplicada, CINBIO, Universidade de Vigo, 36310 Vigo, Spain; uxia.calvino.barreiro@uvigo.es (U.C.); luis.lugo@uvigo.es (L.L.); 2 Área de Máquinas e Motores Térmicos, Escola de Enxeñería Industrial, Universidade de Vigo, 36310 Vigo, Spain; jseara@uvigo.es; 3 Centro Universitario de la Defensa en la Escuela Naval Militar, Plaza de España, s/n, 36920 Marín, Spain; 4 Institut für Luft-und Kältetechnik Dresden, Bertolt-Brecht Allee 22, 01309 Dresden, Germany; matthias.buschmann@ilkdresden.de

**Keywords:** heat transfer coefficients, convection, thermophysical properties, aqueous nanofluid, graphene nanoplatelets

## Abstract

The thermal properties of graphene have proved to be exceptional and are partly maintained in its multi-layered form, graphene nanoplatelets (GnP). Since these carbon-based nanostructures are hydrophobic, functionalization is needed in order to assess their long-term stability in aqueous suspensions. In this study, the convective heat transfer performance of a polycarboxylate chemically modified GnP dispersion in water at 0.50 wt% is experimentally analyzed. After designing the nanofluid, dynamic viscosity, thermal conductivity, isobaric heat capacity and density are measured using rotational rheometry, the transient hot-wire technique, differential scanning calorimetry and vibrating U-tube methods, respectively, in a wide temperature range. The whole analysis of thermophysical and rheological properties is validated by two laboratories. Afterward, an experimental facility is used to evaluate the heat transfer performance in a turbulent regime. Convective heat transfer coefficients are obtained using the thermal resistances method, reaching enhancements for the nanofluid of up to 13%. The reported improvements are achieved without clear enhancements in the nanofluid thermal conductivity. Finally, dimensionless analyses are carried out by employing the Nusselt and Péclet numbers and Darcy friction factor.

## 1. Introduction

Over the past decade, energy has become an essential resource; demand for it is continuously increasing due to the rising population, industrialization and urbanization. According to the Current Policies Scenario in the World Energy Outlook [1], energy demand rises by 1.3% each year up to 2040. The key to decreasing the impact of the rising energy demand lies in the reduction of emissions by means of renewable energies and the development of thermal systems with higher energy performances.

A significant advance in heat transfer systems has been with passive methods that involve no direct external power application, and active schemes, which entail the application of external power [2]. Some of the passive methods used to enhance the performance of heat transfer devices are twisted tape inserts [3], or the inclusion of fins [4] or baffles [5], while some of the active methods are the use of ultrasounds [6] or the application of electrodynamic techniques [7]. Among the passive methods, the use of nanofluids to enhance heat transfer processes has proved to be a promising line of research [8].

The primary limitation on heat transfer fluids’ further improvement of the heat transfer performance is their restricted thermal conductivity. The dispersion of nano-sized particles in widely-used working fluids has been considered as a prospective solution to this limitation [9,10,11]. The most common materials used as nanoadditives are carbon allotropes, metals, metal oxides, carbides or nitrides [12,13,14,15,16]. Regarding base fluids, different heat transfer fluids such as water [17,18,19], ethylene glycol [20,21,22], mixtures of the two [23,24,25], propylene glycol [26], engine oils or refrigerants have been commonly selected [27,28,29].

However, obtaining new heat transfer fluids with higher thermal conductivity should not be the only objective. Regarding practical applications, turbulent forced convection is the most common heat transfer process used in industry and the viscosity of the used fluid plays a critical role, directly affecting the pumping power consumption of the system. Therefore, it is an important matter that should be taken into account for performance improvement. Otherwise, as is well-known, water is one of the most commonly used base fluids when designing nano-enhanced fluids [30,31,32] since it presents a noticeable thermal conductivity and low viscosity. Nanoadditives that have shown great potential in improving heat transfer enhancement are carbon allotropes, such as graphite, diamond, nanotubes or nanohorns [33,34,35]. These nanoadditives present thermal conductivities several orders of magnitude higher than those of the base fluids, and important improvements are achieved at low concentrations [36]. Among them, graphene and carbon nanotubes entail the highest thermal conductivity values. In particular, graphene derivatives stand out as one of the most widely used nanoadditives due to their extraordinary thermal, mechanical and electrical properties [37]. A multi-layered structure, commercially known as graphene nanoplatelet (GnP), partially preserves the properties of the single-layer graphene, while its fabrication cost is significantly lower. Otherwise, GnPs are hydrophobic, which hinders their use as nanoadditives in aqueous media. Functionalization of the material by chemical routes is required (reductions, oxidations or coverings) in order to achieve an equilibrium between the base fluid and the nano-sized particles and compose stable suspensions [38,39].

The heat transfer performance of graphene nanoplatelets in water has been experimentally analyzed in the literature [38,40,41,42,43,44,45,46]. Yarmand et al. [38] analyzed GnP-Ag nanofluids, finding improvements in effective thermal conductivity and heat transfer efficiency compared to the base fluid. The maximum reported enhancement of the Nusselt number compared to distilled water was 32.7%. Arshad et al. [40] observed the thermal and hydrodynamic performance of water-based GnP nanofluids at a 10 wt% nanoadditive concentration, in comparison with distilled water, upon an integral fin heat sink. The maximum convective heat transfer enhancement was 23.9%. Arzani et al. [41] experimentally measured and numerically simulated the convective heat transfer coefficient of water-based tetrahydrofurfuryl polyethylene glycol-treated GnP nanofluids at various weight concentrations in an annular heat exchanger. The maximum enhancement with regard to the base fluid was 25.6%. Yarmand et al. [42] experimentally measured thermo-physical properties and determined the convective heat transfer coefficients of functionalized graphene nanoplatelets aqueous nanofluids. The measurements were made for different weight percentages of nanoadditives (0.02, 0.06 and 0.1 wt%), reaching a maximum enhancement at an overall heat transfer coefficient of up to 19.7%. The thermal conductivity and viscosity of graphene nanoplatelets in distilled water, at concentrations from 0.025 to 0.1 wt%, were measured by Sadeghinezhad et al. [43]. The effects of the concentrations of nanoparticles and heat flux on the enhancement of the heat transfer in a horizontal stainless-steel tube were assessed, obtaining Nusselt number increases of up to 83%. Mehrali et al. [44] experimentally obtained the thermophysical properties of GnP nanofluids at various temperatures, specific surface areas and concentrations (0.025 to 0.1 wt%) in order to calculate the convective heat transfer coefficient of the samples. Higher convective heat transfer coefficients of up to 200% were found for the GnP nanofluids when compared to distilled water. Agromayor et al. [45] analyzed the heat transfer enhancement of aqueous nanofluids. Convective heat transfer coefficients and pressure drops at various mass concentrations of functionalized GnPs, from 0.25 to 1.0 wt%, were obtained; maximum enhancements of 32% in heat transfer coefficients were assessed. Fares et al. [46] studied the convective heat transfer of graphene nanofluids over a vertical shell and tube heat exchanger. Different nanoparticle concentrations, inlet temperatures and flow rates were analyzed, showing a maximum enhancement in the convective heat transfer coefficient of 29% when using 0.2% graphene/water nanofluids.

Nevertheless, comprehensive studies are missing that analyze the entire set of variables affecting the fluid and that influence the application of a graphene-based aqueous nanofluid as heat transfer fluid. In order to completely understand these enhanced heat transfer fluids, broad studies including the whole nanofluid evaluation (design, long-term stability, rheological and thermophysical profiles and convective heat transfer performance) are required. In addition, double-checked experimental properties obtained by different laboratories and equipment comprise the recommended methodology in the nanofluid literature to source reliable data with the aim of drawing far-reaching conclusions.

Therefore, the purpose of this work is to evaluate a nanofluid consisting of carbon-based chemically modified GnP dispersed in water at 0.50 wt% for convective heat transfer applications where the heat transfer occurs at turbulent flow. Initially, the stability of dispersion is measured using zeta potential. Then, the nanofluid’s rheological and thermophysical profiles are assessed by means of different methods available in two laboratories with the aim of sourcing conclusive data. Subsequently, the convective heat transfer coefficients and pressure drops are evaluated using a heat pipe. Finally, a dimensionless analysis of the Nusselt and Péclet numbers and Darcy friction factor is carried out.

## 2. Materials and Methods

### 2.1. Materials and Stability Characterization

Polycarboxylate chemically modified graphene nanoplatelet (P-GnP) nanopowder was dispersed in water at 0.50 wt% following a two-step procedure. The P-GnP nanoadditive was provided by NanoInnova Technologies (Madrid, Spain), while the water was obtained by a Milli-Q 185 Plus system from Millipore (Watford, United Kingdom) with a resistivity of 18.2 MΩ·cm. The quantities of each component were weighed in a CPA225 analytical laboratory balance from Sartorius AG (Göttingen, Germany) with 1·10^−4^ g uncertainty. Afterwards, the dispersions were submerged in an ultrasonic bath from JP Selecta (Barcelona, Spain) for 240 min at 20 kHz frequency and 200 W power.

**Figure 1:**
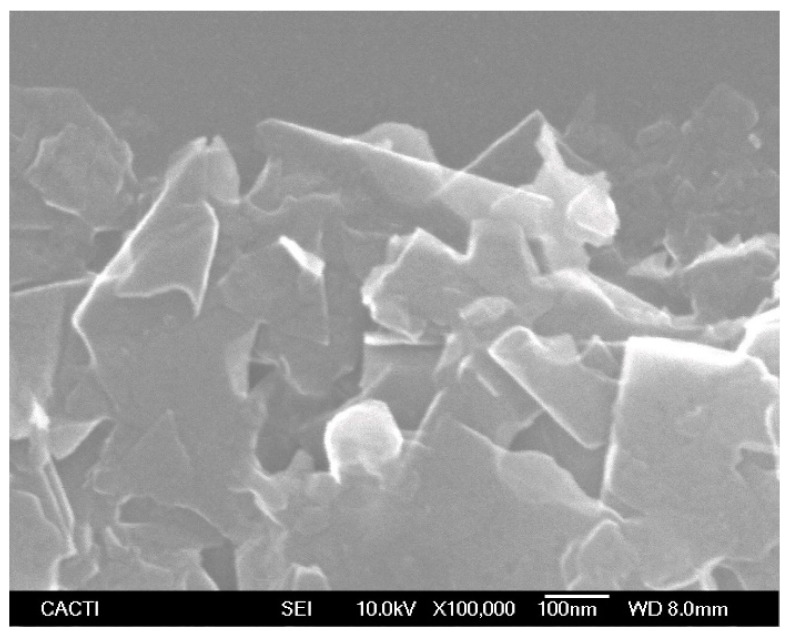
SEM image of P-GnP nanopowder at 100,000× magnification.

A scanning electron microscopy (SEM) analysis of the dry nanopowder was developed using a field emission gun JSM-6700F from JEOL (Tokyo, Japan), coupled with an Yttrium Aluminum Garnet detector and operating at an accelerator voltage of 10.0 kV. A drop of nanopowder was dispersed in analytical-grade methanol and dried over the silica support for the analysis. Figure 1 contains an SEM image of the P-GnP nanopowder showing several platelet-shaped nanostructures with their characteristic straight and regular edges. Based on previous atomic force microscopy and electron miscopy studies [26,30], it was known that these nanoplatelets consist of 5–10 stacked graphene layers, each 2–18 nm in height, while their lengths and widths can reach 500–550 nm. The current SEM analysis confirmed this information. Previous energy-dispersive X-ray spectroscopy and elemental analyses confirmed the presence of carbon, oxygen and potassium in this commercial nanopowder [9,47].

The nanofluid stability was evaluated by a zeta potential analysis carried out using a Zetasizer Nano ZS from Malvern Instruments (Malvern, UK). The measurements were made using DTS1070 model cells at 298.15 K. A zeta potential value of around −54 mV was obtained, which is symptomatic of good dispersion stability with low settling, according to the literature criteria [9,10,48]. The experimental pH value of the dispersion was 4.9, obtained using a Sension + PH3 pH-meter from Hach (Loveland, CO, USA), coupled with a 5010-code electrode.

### 2.2. Experimental Methods

The thermophysical and rheological properties of the nanofluid needed in later analyses of the heat transfer and flow behavior were experimentally measured in a temperature range from 283.15 to 323.15 K in Universidade de Vigo (UVigo) and Institut für Luft-und Kältetechnik Dresden (ILK) laboratories. Experimental densities, ρ, and isobaric heat capacities, *c_p_*, were determined using the oscillating U-tube technique and the quasi-isothermal temperature-modulated differential scanning calorimetry methods, respectively, in both laboratories. Rheological characterizations to determine dynamic viscosities, *η*, were carried out using the rotational rheometry (UVigo) and oscillating piston (ILK) methods. Finally, thermal conductivities, *k*, were determined using a commercial device based on the transient hot-wire method (UVigo) and using an own-developed ring-gap apparatus [49,50] based on a steady-state method (ILK). 

**Figure 2:**
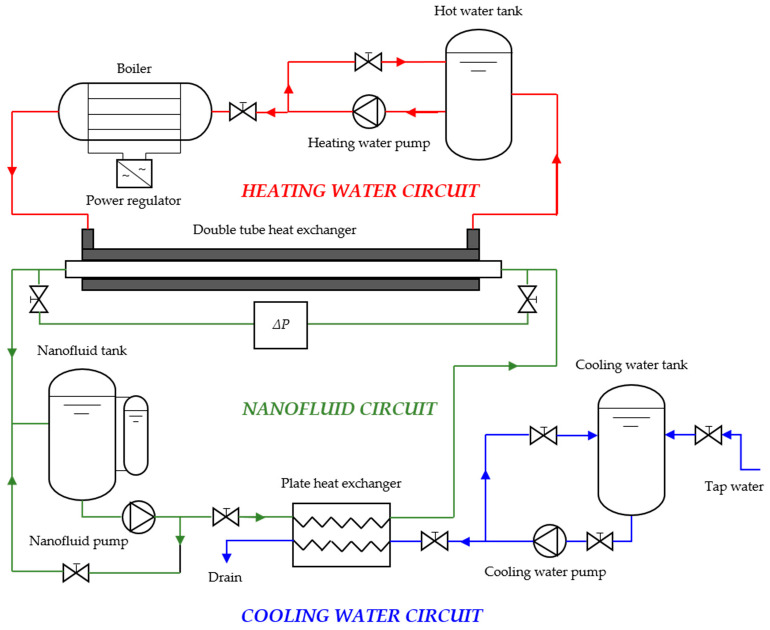
Scheme of the experimental test rig used to obtain convective heat transfer coefficients and pressure drops.

Figure 2 shows the outline of the experimental facility where the convective heat transfer coefficients, *h*, and pressure drops, Δ*P*, were obtained. It is structured in three hydraulic circuits: the nanofluid and heating water circuits (closed loop) and a cooling water circuit (open loop). The key to this experimental device is an isolated double-tube heat exchanger made of stainless steel (AISI 316 L). Energy is transferred from the annular section (where the heating water flows) to the inner pipe (where the nanofluid flows in a counter-current). The inner tube presents an internal diameter, *d_1_*, of 0.008 m, and an external diameter, *d_2_*, of 0.010 m, while the outer tube has an internal diameter, *d_3_*, of 0.015 m. Meanwhile, the heat exchanger’s effective lengths are 0.93 m for heat exchange, *L_h_*, and 1.18 m for pressure drop, L_Δ*P*_. In previous works, detailed descriptions of the mentioned test rig have been reported [29,51,52].

Different tests sweeping several temperatures as well as various turbulence levels were performed. These tests determined the volumetric flow rates and the average temperature of the cold fluid (nanofluid) and hot fluid (water) over the double-tube heat exchanger. Two average temperatures were considered for the cold fluid (base fluid or nanofluid), 308.15 and 318.15 K, while the hot fluid (water) remained 15 K above these values. To analyze the influence of turbulence, the cold fluid flow rate was varied from 0.3 to 0.7 m^3^·h^−1^, with a 0.1 m^3^·h^−1^ step, while the hot fluid flow rate was maintained at 0.7 m^3^·h^−1^ for all tests.

The double-checked experiment’s rheological and thermophysical properties and the data measured at the experimental facility for water and the nanofluid are used herein to analyze the convective heat transfer performance and fluid flow behavior. The convective heat transfer coefficients of the hot fluid, *hf*, (water) were obtained using the Gnielinski [53] correlations for a fully developed turbulent flow through annular ducts. Then, those coefficients for the cold fluid, *cf*, (base fluid or nanofluid) were determined using the thermal resistances method. This method considers the transmitted heat from the hot fluid to the steel wall by convection, *R_hf_*, crosswise the steel wall, *s*, by conduction, *R_s_*, and from the wall to the cold fluid (base fluid or nanofluid) by convection, *R_cf_*. The sum of the three resistances is the total thermal resistance, *R_T_*, which can also be obtained by dividing the logarithmic mean temperature difference, Δ*T_ML_*, by the exchanged heat flux, $\dot{Q}$ [54],

(1) $$R_{T}\;=R_{hf}+R_{s}+R_{cf}\;=\;\frac{\Delta T_{ML}}{\dot{Q}}$$

(2) $$R_{hf}=\;\frac{1}{\pi \cdot d_{2}\cdot L_{h}\cdot h_{hf}}$$

(3) $$R_{s}=\;\frac{\ln\left(d_{2}/d_{1}\right)}{2\pi \cdot k_{s}\cdot L_{h}}$$

(4) $$R_{cf}=\;\frac{1}{\pi \cdot d_{1}\cdot L_{h}\cdot h_{cf}}$$

where $h_{hf}$ is the hot fluid (water) convective heat transfer coefficient, $h_{cf}$ is the cold fluid (base fluid or nanofluid) convective heat transfer coefficient, $L_{h}$ is the effective length of heat exchange, *d_1_* is the internal diameter of the inner tube, *d_2_* is the external diameter of the inner tube and $k_{s}$ is the thermal conductivity of the steel.

## 3. Results

### 3.1. Thermophysical and Rheological Properties

Thermophysical properties and a rheological profile of the nano-enhanced fluid were experimentally determined by both laboratories. Similar nanofluid densities were obtained (within an AAD% of 0.1%). Increases in the experimental density values—due to the dispersion of nanoplatelets in the base fluid—of up to 0.2% were reached. In addition, density decreases for both the nanofluid and base fluid of 1% were observed when the temperature rose from 293.15 to 323.15 K.

**Figure 3:**
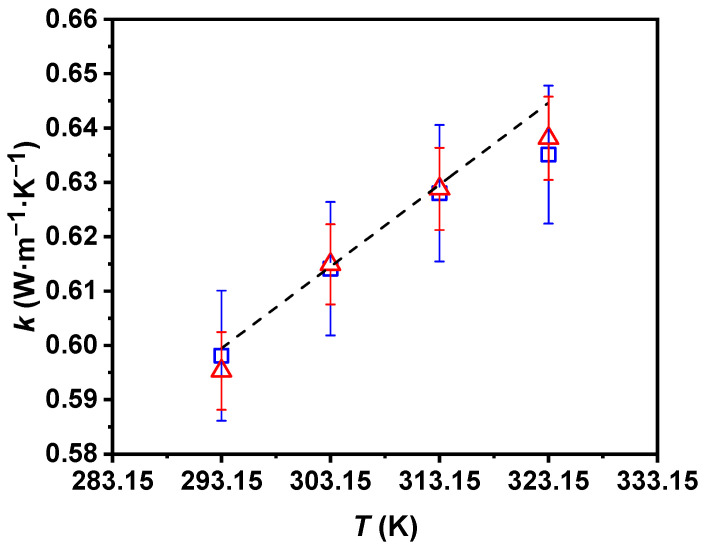
Experimental thermal conductivity, *k*, of 0.50 wt% P-GnP/W nanofluid (UVigo laboratory, □; ILK laboratory, △) together with the literature *k* of water (- - -) [55] as a function of temperature, *T*. Error bars indicate expanded uncertainty (*k* = 2).

Regarding the thermal conductivity of water, similar experimental data to that of the literature were obtained, with a maximum deviation between the literature and experimental values of less than 1%. The experimental thermal conductivity of the nanofluid and values from the literature for water are shown in Figure 3 as a function of temperature. Regarding the measured values, there was also good agreement between the UVigo and ILK results for the nanofluid, with the compatible thermal conductivity values for all analyzed temperatures deviating by less than 0.5%. The thermal conductivity values increased by up to 6.2% with the rising temperature when taking the value at the lowest temperature as a reference.

**Figure 4:**
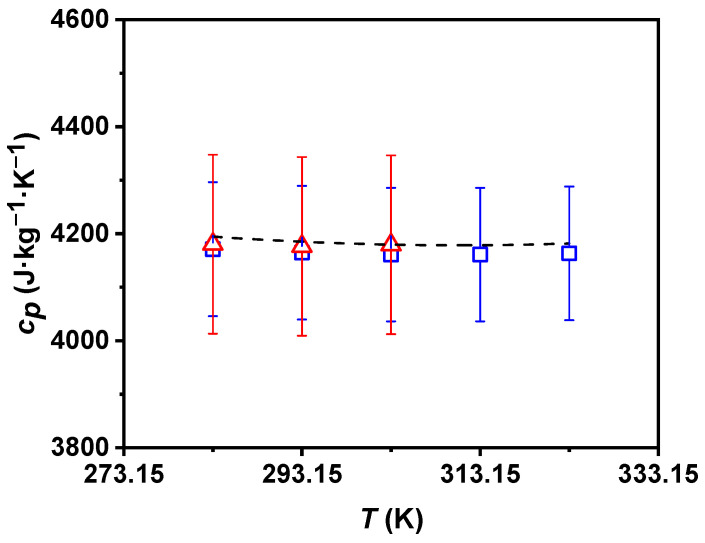
Experimental isobaric specific heat capacity, *c_p_*, of 0.50 wt% P-GnP/W nanofluid (UVigo laboratory, ☐; ILK laboratory, △) and literature *c_p_* of water (- - -) [55] as a function of temperature, *T*. Error bars indicate expanded uncertainty (*k* = 2).

With the aim of achieving a further analysis of the studied fluids, the isobaric heat capacities were also experimentally determined by both laboratories. The experimental *c_p_* values for water were in good agreement with those reported in the literature, with the maximum deviation being less than 1%. The obtained values for the 0.50 wt% P-GnP/W nanofluid are shown in Figure 4; taking into account the combined experimental uncertainty corresponding to the UVigo and ILK devices, good agreement between laboratories can be observed. It is also noteworthy that the tested nanofluid presents similar isobaric-specific heat capacity—temperature dependences to the employed base fluid. The specific heat capacity values of the P-GnP/W nanofluid are smaller than those of the base fluid, as expected, due to the lower heat capacity of solids [10]. Nanoadditive loading causes decreases of up to 0.4%.

**Figure 5:**
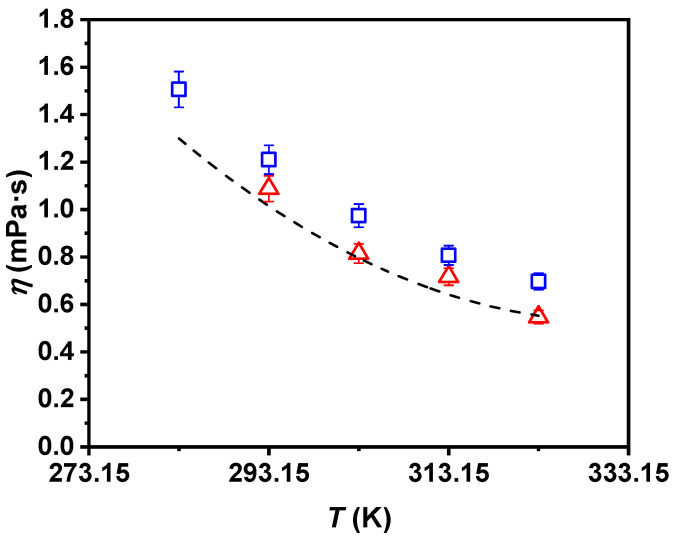
Experimental dynamic viscosity, *η*, of 0.50 wt% P-GnP/W nanofluid (UVigo laboratory, ☐; ILK laboratory, △) and *ƞ* of water (- - -) [55] as a function of temperature, *T*. Error bars indicate expanded uncertainty (*k* = 2).

Regarding the rheological profile of the studied fluids, Figure 5 shows the experimental dynamic viscosity values of the nanofluid and the literature’s values of the base fluid over the entire temperature range. An average deviation of 2% was obtained between the experimental and literature values for water [55]. It can be observed that the UVigo and ILK experimental viscosity results obtained for the 0.50 wt% P-GnP/W nanofluid agree, according to the experimental uncertainty of both devices. Decreases in the nanofluid’s dynamic viscosity with increasing temperature reached 116% over the whole analyzed range. As is well-known, the higher the concentration of solid nanoparticles in a liquid, the greater the resistance to flow due to the increase in friction inside it. This increment with the nanoadditive loading, in terms of dynamic viscosity, was around 23%. A Newtonian behavior for the nanofluid was proved in both laboratories with congruent dynamic viscosity data between both set points at a maximum deviation of less than 10%.

### 3.2. Heat Transfer Performance and Fluid Flow Behavior

**Figure 6:**
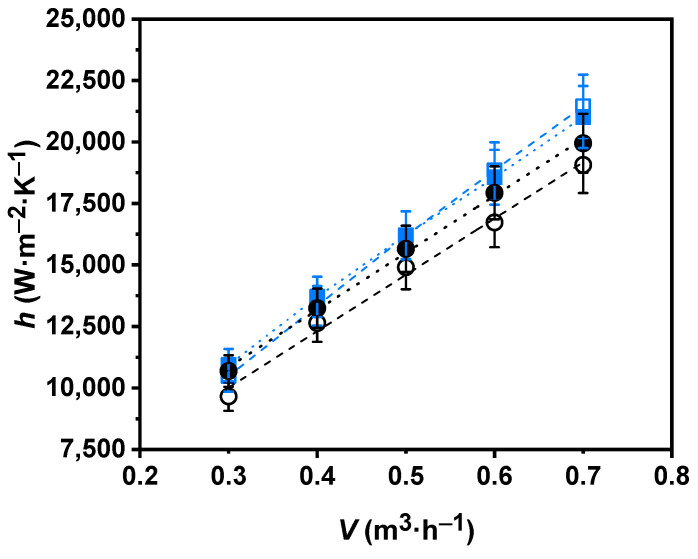
Experimental convective heat transfer coefficients, *h*, for water (black) and 0.50 wt% P-GnP/W nanofluid (blue) as a function of volume flow rate, *V*, at 308.15 K (○, □) and 318.15 K (●, ■). Error bars indicate expanded uncertainty (*k* = 2).

In order to verify the accuracy of the measurements, the results obtained for water flowing through the nanofluid circuit of the test rig were compared with the theoretical values determined from the well-known Gnielinski correlations in pipe flow [47]. The obtained average deviation was less than 10.5%. It should be noted that the experimental data obtained by the experimental facility were verified beforehand for water [30,42,45]. Convective heat transfer coefficients of the nanofluid and the base fluid at 308.15 and 318.15 K for different analyzed flows are shown in Figure 6. Both the P-GnP/W nanofluid and water increased their convective heat transfer coefficients as the temperature and flow rates rose. Increases of up to 10.7% were obtained by raising the temperature from 308.15 to 318.15 K. Regarding the behavior of the convective heat transfer coefficients with the rising level of turbulence, increases of 105% and 92.2% for the nanofluid and 97.5% and 86.6% for water were reached, at 308.15 and 318.15 K, respectively. Moreover, maximum enhancements with the nanoadditive loading of up to 13% were obtained in the analyzed flow rate range.

It should be noted that the enhancement in convective heat transfer coefficients was obtained for a sample with no clear improved thermal conductivity. This means that the turbulent mixing phenomenon within the nanometric dispersion provides a superior benefit in terms of the heat transfer performance than the modified intrinsic transport property of the two-phase sample.

**Figure 7:**
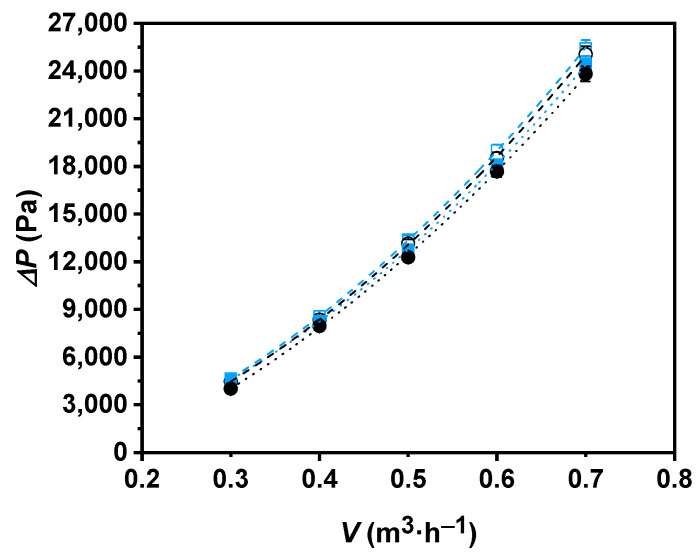
Experimental pressure drops, Δ*P*, for water (black) and 0.50 wt% P-GnP/W nanofluid (blue) as a function of volume flow rate, *V*, at 308.15 K (○, ☐) and 318.15 K (●, ■). Error bars indicate expanded uncertainty (*k* = 2).

Pressure drops for the base fluid and nanofluid through the concentric tube heat exchanger were also measured. Friction losses inside the double tube increased with the level of turbulence; this phenomenon is reflected in Figure 7, where higher pressure drops are shown with the increasing flow rate. High pressure drops entail higher pumping powers, which affects the total energy efficiency. Thus, excessive increases in the pressure drop of the working fluid may involve the frustration of a new potential competitive fluid. Values of up to 4.5 times higher for the P-GnP/W and 4.9 times higher for water were reached within the analyzed flow rate range. Figure 7 also shows tolerable pressure drop increases in the nanofluid versus the base fluid that reach 6%.

The pressure drop’s dependence on temperature can be also observed in Figure 7. According to the dynamic viscosity decrease at higher temperatures, smaller pressure drop values were found for both fluids in the highest temperature test. The decrease in the pressure drop reached 4.8% for the P-GnP/W nanofluid and 22% for water when the temperature was raised from 308.15 to 318.15 K.

### 3.3. Dimensionless Analysis

The structural parameters of the concentric tube heat exchanger of the test rig were used to obtain convective heat transfer coefficients and pressure drops. In order to extend the scope of the study to other facilities, a dimensionless analysis was proposed from the obtained experimental values. The employed dimensionless numbers in this analysis were the Nusselt number, *Nu*, (Equation (5)); Reynolds number, *Re*, (Equation (6)); Péclet number, *Pe*, (Equation (7)); and Darcy friction factor, *f*, (Equation (8)):

(5) $$Nu\;=\;\frac{h_{cf}\cdot d_{1}}{k_{cf}}$$

(6) $$Re\;=\;\frac{\rho_{cf}\cdot {\dot{v}}_{cf}\cdot L_{h}}{\eta_{cf}}$$

(7) Pe = Re·Pr

(8) $$f\;=\;\frac{\pi^{2}\cdot d_{1}{}^{5}\cdot \Delta P_{cf}}{\;8\cdot L_{\Delta p}\cdot \rho_{cf}\cdot {\dot{v}}_{cf}^{2}}$$

where $k_{cf}$*,* $\rho_{cf}$, ${\dot{v}}_{cf}$ and $\eta_{cf}$ are the thermal conductivity, density, volume flow rate and dynamic viscosity of the cold fluid, respectively; $L_{h}$ is the effective length of the heat exchange; *Pr* is the Prandtl number $\left(Pr\;=\;\frac{cp_{cf}\cdot \eta_{cf}}{k_{cf}}\right)$; *d_1_* is the internal diameter of the inner tube of the heat exchanger; $\Delta P_{cf}$ is the experimental pressure drop of the cold fluid; and $L_{\Delta P}$ is the effective length of the pressure drop.

**Figure 8:**
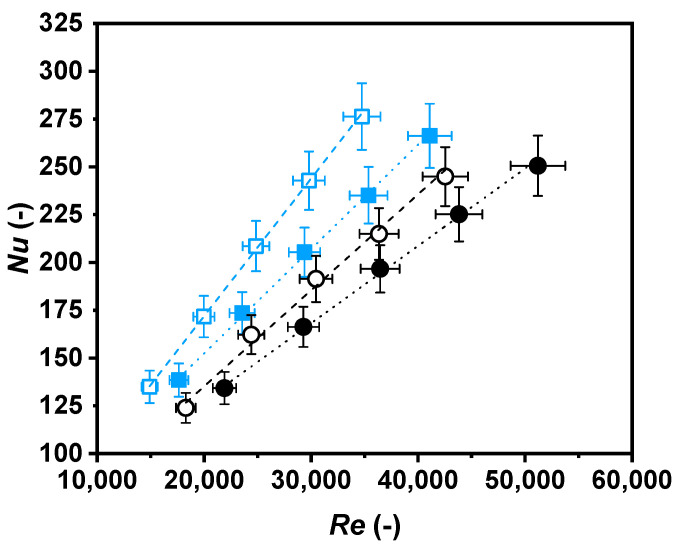
Nusselt number as a function of Reynolds number for water (black) and nanofluid (blue) at 308.15 K (○, ☐) and 318.15 K (●, ■).

**Figure 9:**
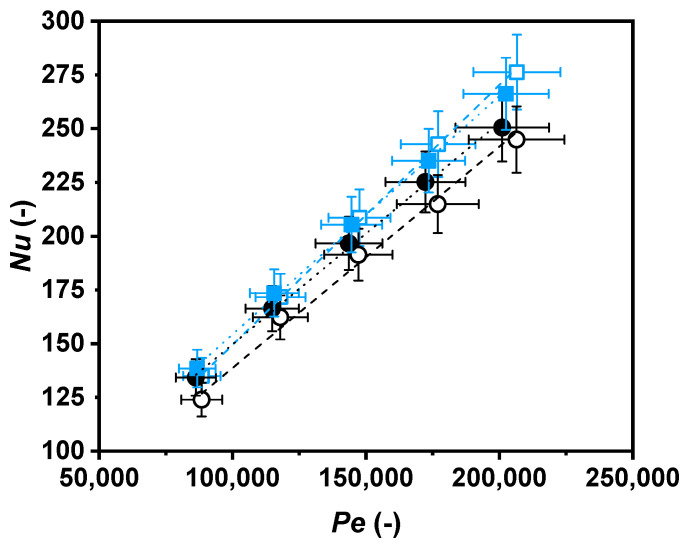
Nusselt number as a function of Péclet number for water (black) and nanofluid (blue) at 308.15 K (○, ☐) and 318.15 K (●, ■).

**Figure 10:**
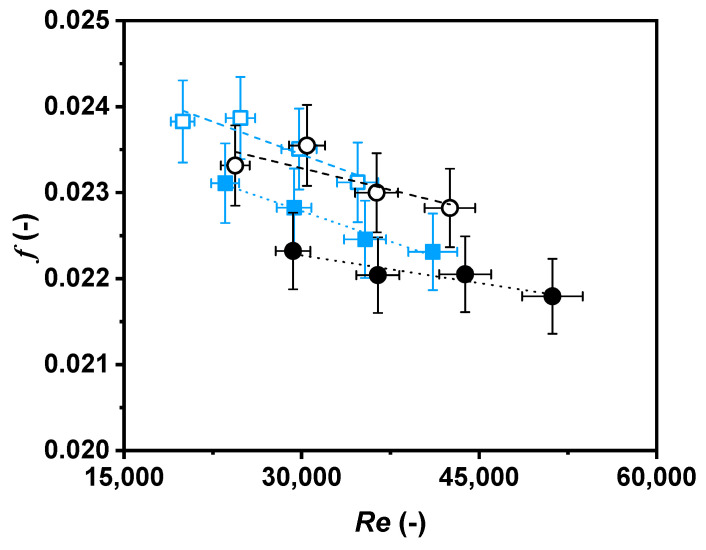
Darcy friction factor as a function of Reynolds number for water (black) and nanofluid (blue) at 308.15 K (○, □) and 318.15 K (●, ■).

Hereon, Nusselt numbers as a function of Reynolds numbers (Figure 8), Nusselt numbers as a function of Péclet numbers (Figure 9), and Darcy friction factors as a function of Reynolds numbers (Figure 10) are shown for the nanofluid and the base fluid at two temperatures, 308.15 K and 318.15 K.

A rise in the Nusselt number with the increase in the Reynolds number can be observed due to greater turbulence. The nanofluid’s Nusselt number increased when compared to the Reynolds number by around 105% and 92% at 308.15 K and 318.15 K, respectively. These values decreased to 97% and 86% at 308.15 K and 318.15 K, respectively, for the base fluid. Comparing both samples at the same level of turbulence, a higher ratio of convective to conductive heat transfer was found for the nanofluid. As an example, the Nusselt number was around 29% higher for the nanofluid than for the base fluid at 308.15 K, and 23% at 318.15 K, for a Reynolds number of 30,000.

Higher Nusselt numbers for the nanofluid than for the base fluid at the two temperatures are shown in Figure 9 as a function of the Péclet number. As an example, these enhancements reached 9.2% at 308.15 K and 3.9% at 318.15 K for a Péclet number of 150,000.

The Nusselt number of the nanofluid increased at the same value as the Péclet number due to the higher level of turbulence, as is also illustrated in Figure 9, and due to the higher ratio of momentum diffusion to thermal diffusion. The rise in the Péclet number is an effect of the intensification of the fluid flow rate, which automatically increases the heat transfer of fluids [56].

Darcy friction factors, *f*, are shown in Figure 10 for both the nanofluid and base fluid. The obtained *f* values and their Reynolds number dependence agree with the Moody diagram for turbulent flow through smooth pipes, which are the operating conditions of this work. It is noteworthy that Darcy friction factor values of the nanofluid remained higher than those of the base fluid at both temperatures, in accordance with the experimental pressure drop shown in Figure 7. As an example, the Darcy friction factor was around 0.7% higher for the nanofluid than for the base fluid at 308.15 K, and 2.3% at 318.15 K, for a Reynolds number of 30,000.

## 4. Conclusions

The thermophysical and rheological properties for a new polycarboxylate chemically modified GnP 0.50 wt% aqueous nanofluid were experimentally determined by two laboratories, showing a good agreement. Newtonian behavior was detected for the tested nanofluid, with dynamic viscosity increases of up to 23%. As regards the base fluid, the density of the nanofluid increased by 0.2%, while the isobaric heat capacity decreased by 0.4%. Thermal conductivities for the base fluid and nanofluid showed similar values.

In addition, experimental convective heat transfer coefficients and pressure drops were determined. Enhancements of up to 13% for the coefficients and moderate pressure drop increases of less than 6% were achieved for the proposed nanofluid. A dimensionless analysis concluded that the nanofluid shows superior Nusselt numbers to those of the base fluid for the same flow rate. These improved Nusselt numbers entail a noticeable enhancement of the convective heat transfer performance. It is noteworthy that this enhancement was achieved without clear increases in the thermal conductivity of the nanofluid with regard to the base fluid. This confirms that the turbulent mixing phenomenon within the nanometric dispersion provides a superior benefit in terms of the heat transfer performance than the modified intrinsic transport property of the two-phase sample. Moderate increases in the Darcy friction factor values of the nanofluid were obtained in relation to those of the base fluid, entailing low rises in pumping power consumption. Therefore, the tested nanofluid is a good potential candidate for a nano-enhanced working fluid for industrial forced-convective heat transfer processes.
